# Field-Based Health-Related Physical Fitness Tests in Children and Adolescents: A Systematic Review

**DOI:** 10.3389/fped.2021.640028

**Published:** 2021-03-05

**Authors:** Adilson Marques, Duarte Henriques-Neto, Miguel Peralta, João Martins, Fernando Gomes, Stevo Popovic, Bojan Masanovic, Yolanda Demetriou, Annegret Schlund, Andreas Ihle

**Affiliations:** ^1^CIPER, Faculty of Human Kinetics, University of Lisbon, Lisbon, Portugal; ^2^ISAMB, University of Lisbon, Lisbon, Portugal; ^3^Faculty of Human Kinetics, University of Lisbon, Lisbon, Portugal; ^4^Faculty for Sport and Physical Education, University of Montenegro, Niksic, Montenegro; ^5^Department of Sport and Health Sciences, Technical University of Munich, Munich, Germany; ^6^Cognitive Aging Lab, Department of Psychology, University of Geneva, Geneva, Switzerland; ^7^Center for the Interdisciplinary Study of Gerontology and Vulnerability, University of Geneva, Geneva, Switzerland; ^8^Swiss National Centre of Competence in Research LIVES – Overcoming Vulnerability: Life Course Perspectives, Lausanne and Geneva, Switzerland

**Keywords:** body composition, cardiorespirarory fitness, fitness testing, musculoskeletal, physical education, vulnerability

## Abstract

Physical fitness (PF) is a multi-component construct and a biomarker of health. Worse PF is related to vulnerability and predicts worse academic achievements. Thus, assessing PF is important to monitor health in youth. This systematic review aimed to identify and inform physical education, health professionals and entities about existing PF batteries and field-tests that can be used in school settings. A comprehensive literature search was carried out in five electronic databases (Academic Search Complete, Education Resources Information Center, PubMed, Scopus, and Web of Science) to identify PF battery protocols that can be carried out in the school setting. Overall, 24 PF batteries were identified. Regarding the PF components assessed, only cardiorespiratory fitness and upper body strength were contemplated in all batteries. Middle-body strength and lower body strength were presented in most batteries (21 and 19 of 24, respectively). Agility (16 of 24) and body composition (16 of 24) were also considered in several batteries, although to a lesser extent. Flexibility (14 of 24) and speed (12 of 24) were the PF components less represented in the batteries. Among the 24 identified PF batteries, 81 PF tests assessing the different PF components were encountered. The advances in the PF field-based assessment in school settings and health in youth resulted in the amplification of the number of existing batteries. Considering the connection between PF and health and the opportunity that the school setting provides to assess fitness in children and adolescents, there is a need for standardization and a consensus of PF assessments in this specific setting.

## Introduction

Physical fitness (PF) is a multi-component construct and a biomarker of health (1, 2). Worse PF is related to vulnerability (3) that can negatively affect human development, such as cognitive functioning (4, 5). This has important consequences children and adolescents. For instance, it has been shown that worse PF predicts substantially reduced improvements in academic achievement over time (6). PF is influenced by genetic and external factors (7). The genetic heritage has an essential role in trainability and describes the magnitude of the physiologic response to physical stress (2, 8). External factors such as regular PA, sleep, nutrition also have an impact on PF components (9–11). Assessing PF through specific and validated test protocols allows monitoring the biological and physiological adaptations that are achieved through natural development or training (12). Health-related PF components include body composition measures (i.e., body mass index [BMI], waist circumference), cardiorespiratory fitness (CRF), muscular fitness, speed, agility, balance, and coordination (13, 14). These components have been consistently associated with indicators of obesity, cardiovascular health, metabolic health, bone health, and mental health (1).

Assessing PF reflects the impact of genetic and environmental factors on health-related PF components and consequently on health indicators (15). In light of this, assessing PF is a simple, safe, and low-cost tool that allows examining several health indicators. Based on the PF level of children, pedagogical, and public health strategies and policies can be developed. However, to correctly and accurately assess PF, the validity, reliability, and feasibility of PF assessment tools are essential. This is especially true when health and government entities aim to monitor a variety of health indicators in local, regional, national, or worldwide populations to guide policy actions.

Previous systematic reviews identified a large number of test batteries available worldwide to test children's and adolescents' PF levels (16–18). These reviews showed that different tests address different components of fitness such as cardiorespiratory fitness, musculoskeletal fitness, body composition, and central body fat. Although the selected tests are extensively used and recognized, they do not determine all physical fitness aspects. Moreover, a large number of field-based fitness tests presented in these systematic reviews have limited evidence (16, 18). Furthermore, previous reviews sought to identify physical fitness tests that could be used with children and adolescents. However, some of the contexts identified for the application of some batteries were the sport context. This context is elitist because few children and adolescents practice physical activity in the sports context.

So far no systematic review that provides a summary of all existing fitness test batteries for children and adolescents that can be carried out in the school setting under the specific circumstances of the school (e.g., time constraints, equipment at schools, the scope of testing, costs) has been carried out. Therefore, this systematic review aimed to identify and summarize the existing field-based health-related PF batteries that can be performed in children and adolescents to monitor and improve their health status.

## Methods

Data selection, collection, and analyses were performed following the Preferred Reporting Items for Systematic Reviews and Meta-Analyses (PRISMA) statement (19).

### Search Strategy and Data Sources

Five international databases (Academic Search Complete [ASC], Education Resources Information Center [ERIC], PubMed, Scopus, and Web of Science) were searched for scientific articles published in peer-reviewed journals until the 30th of April 2020 containing PF battery protocols. In each database, a search was conducted taking into account a predefined combination of keywords. The combination of keywords used in each database was the following: “field-based test” OR “fit^*^” OR “physical performance” OR “sport performance” OR “physical condition” OR “aerobic capacity” OR “maximum oxygen consumption” OR “strength” OR “flexibility” OR “motor” OR “endurance” OR “speed” OR “agility” OR “balance” OR “body composition” OR “anthropometry” OR “body mass index” OR “BMI” OR “skinfolds” OR “waist circumference” AND “batter^*^” OR “protocol^*^” OR “assess^*^” OR “valid^*^” OR “reproduct^*^” OR “feasab^*^” OR “measur^*^” AND “adolescent^*^” OR “child^*^” OR “young^*^” OR “school age” OR “school-aged” OR “youth”. The keywords were selected and defined by consensus from all authors. Furthermore, the reference lists of individual studies that reported results or used PF batteries in their methodologies but did not present the protocol were searched for records containing those protocols. Records identified through this method were added as records identified through other sources.

### Inclusion Criteria

This systematic review includes scientific articles from peer-reviewed journals that contained PF battery protocols published until the 30^th^ of April 2020. Only records presenting PF batteries comprising field-based health-related PF tests for children and adolescents that could be performed in the school setting were included. Thus, inclusion criteria were the following: (1) presenting results on the identification, structure, validity, reliability or feasibility of PF batteries, or parts of it (including specific tests), assessing health-related PF components in children and adolescents; (2) containing PF batteries comprising field-based tests that can be performed in the school setting; (3) having a cross-sectional, prospective, observational, experimental, or narrative review study design; (4) being written in English, French, German, Spanish, or Portuguese. Records presenting findings on motor skills, other populations that were not children or adolescents, or not meeting all inclusion criteria were excluded.

### Data Extraction and Selection

The data extraction process was conducted based on PRISMA guidelines (19). After downloading the records from the databases to a reference managing software and integrating further records identified through other sources, duplicates were removed. Two authors (DHN and MP) screened the remaining records for title and abstract to identify studies that met the inclusion criteria. Relevant articles were retrieved for a full read. Then, the two authors reviewed the full text of potential studies, and decisions to include or exclude studies in the review were made by consensus. Disagreements were solved by consensus and, when necessary, a third reviewer served as a judge (AM). Agreement between reviewers was assessed using k statistics (k=0.96) for full-text screening and rating of relevance.

### Data Analysis

Each identified PF battery was entered into a Microsoft Excel (Microsoft Corp., Redmond, Washington, DC, USA) spreadsheet, including information on author and year of publication; country; setting and age range of application; PF components assessed, and the PF tests used for each assessed component. The considered components of PF were body composition, CRF, upper body strength, lower body strength, middle-body strength, speed, agility, and flexibility. Also, a narrative synthesis was performed to describe each field-based health-related PF test in the identified PF batteries.

## Results

### Study Selection

**Figure 1:**
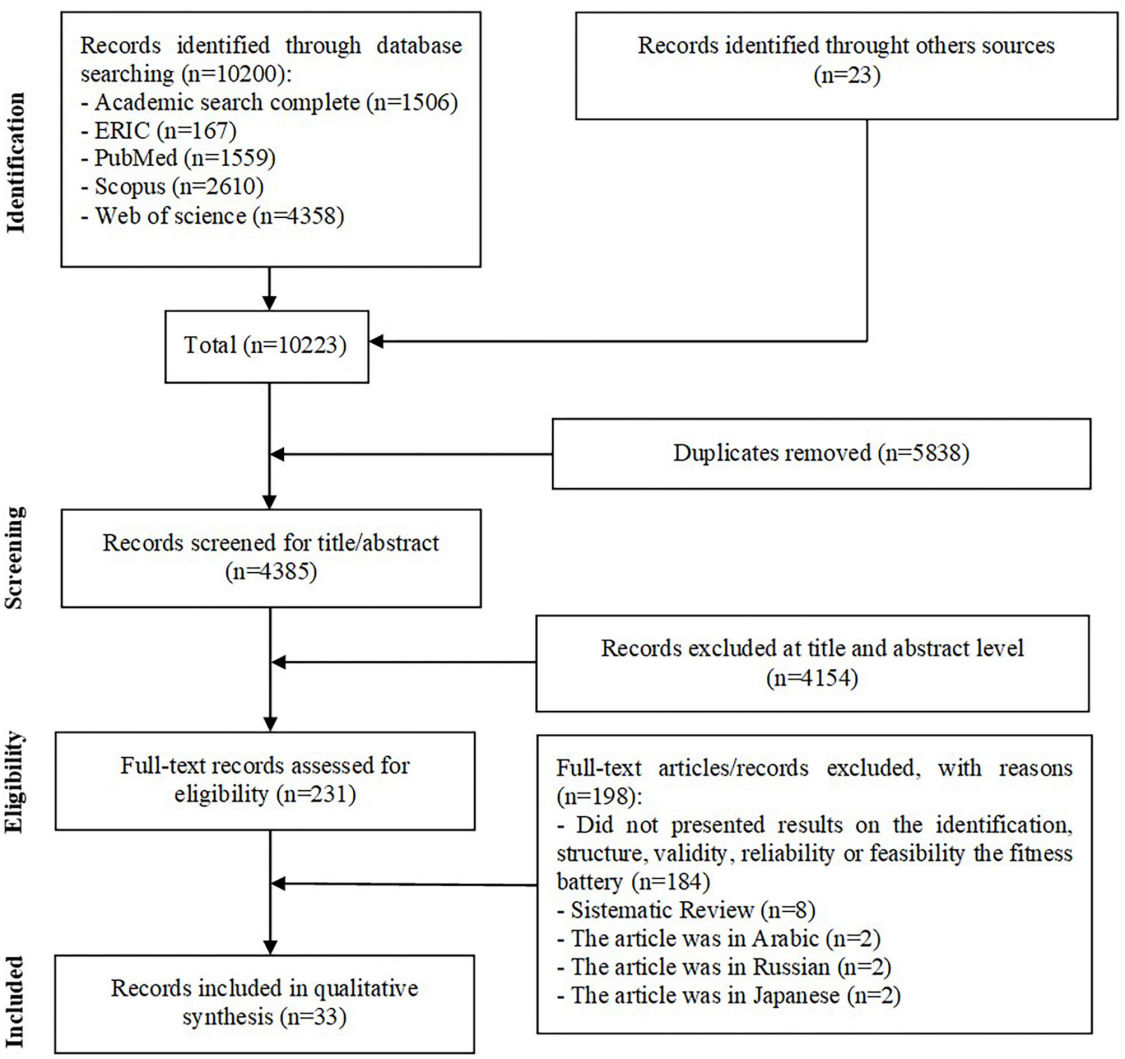
Flow diagram of study selection.

A total of 10223 records (1506 from ASC; 167 from ERIC; 1559 from PubMed; 2610 from Scopus; 4358 from Web of Science; and 23 from other sources) were identified. After removing duplicates (*n*=5,838), 4,385 records were screened based on title and abstract, resulting in 4,154 records excluded. A total of 231 records were assessed for eligibility by full-text reads. Finally, 33 articles matched all inclusion criteria and were included in the qualitative synthesis. The flow chart of records selection is presented in Figure 1.

### Summary of the Identified Physical Fitness Batteries

Table 1 presents a summary of the PF batteries identified in the included records, showing author, year, country, setting, age-range, and test for the following PF components: body composition, CRF, upper body, middle-body and lower body strength, endurance and power, speed, agility, and flexibility. From the 33 included records, 25 PF batteries were identified. Nine PF batteries were from America (six from the United States, two from Canada, one from Brazil) (20–28), nine were from Europe (two from the Czech Republic, one from each of the following countries Norway, Slovenia, Portugal, Italy, France, and Spain, one from the European Union) (15, 23, 24, 27, 28, 30, 32, 41, 42), four were from Asia (one from each of the following countries: Japan, Singapore, China, and Russia) (33, 34, 38, 40), two were from Oceania (one from Australia, one from New Zealand) (22, 36), and one from the Middle East (Bahrain) (31).

**Table 1:** Presentation and summary of the physical fitness batteries identified in the included records.

**Batteries (country); author (year)**	**Setting; age range**	**Physical fitness measurements/tests**											
		**Body composition**	**Cardiorespiratory fitness**	**Upper body**			**Middle-body**		**Lower body**		**Speed**	**Agility**	**Flexibility**
				**Strength**	**Endurance**	**Power**	**Strength**	**Endurance**	**Endurance**	**Power**			
AAHPER (USA); (20)	School and health; 5 to 18 years	None	Half-mile run/walk	None	Bent arm hang; pull-ups	Softball throw	None	Sit-ups	None	Standing broad jump	50-yard dash	4 × 30ft shuttle run	None
AAUTB (USA); (21)	School and sports; 6 to 17 years	None	Shuttle run test (Hoosier-60ft)	None	Modified push-ups; isometric push-ups; bent arm hang	None	None	Sit-ups	Phantom chair	Standing broad jump	50 m dash; 100m dash	4 × 10 m shuttle run	Sit and reach
ACHPER (Australia); (22)	School and health; 9 to 18 years	Height; weight; BMI	PACER	None	None	Basketball throw	None	Sit-ups	None	None	None	None	Sit and reach
ALPHA (Spain); (15)	School, sports, and health; 6 to 18 years	Height; weight; BMI; WC; %BF (skinfolds)	PACER	Handgrip	None	None	None	None	None	Standing broad jump	None	4x10m shuttle run	None
ASSO-FTB (Italy); (23)	School and health; 13 to 17 years	Height; weight; BMI; WC	PACER; 1-mile run / walk	Handgrip	None	None	None	Sit-ups	None	Standing broad jump	None	4x10m shuttle run	None
Bouge (France); (24)	School and health; 6 to 18 years	Height; weight; BMI	Half-mile run / walk; Navette test (20m)	None	None	Basketball throw	None	Curls-ups	None	Standing broad jump	50m dash	10x5m shuttle run	Sit and reach; shoulder stretch
CAHPER-FPT (Canada); (25)	School; 7 to 17 years	None	Half-, 1- and 1.5-mile run / walk; 1000m run	None	Bent harm hang	None	None	Sit-ups	None	Standing broad jump	50m dash; 100m dash	4x10m shuttle run	None
CPAFLA (Canada); (26)	School and health; 15 to 69 years	Height; weight; BMI; WC; HC; waist to hip ratio; %BF (skinfolds)	Step test	None	Handgrip; push-ups	None	None	Modified curl-ups; trunk-lift	None	Standing broad jump	None	None	Sit and reach
EUROFIT (Europe); (27)	School and health; 6 to 18 years	Height; weight and %BF (skinfolds)	PACER; 6 minute run test.	None	Handgrip; bent arm hang	None	None	Sit-ups; trunk-lift	None	Standing broad jump	Plate tapping	10x5m shuttle run	Sit and reach
FITescola (Portugal); (28)	School, sports, and health; 10 to 18 years	Height; weight; BMI; WC; %BF (skinfolds); BIA	PACER; 1-mile run / walk	None	Push-ups	None	None	Sit-ups	None	Standing broad jump; vertical jump	20m and 40m dash	4x10m shuttle run	Sit and reach test; shoulder stretch
FitnessGram (USA); (29)	School, sports, and health; 5 to 17 years	Height; weight; BMI; %BF (skinfolds); BIA	PACER; 1-mile run	None	Push-ups; bent arm hang; pull-ups; modified pull-ups	None	None	Curl-ups	None	None	None	None	Sit and reach; shoulder stretch
Physical Fitness Test Battery (Norway) (30)	School and health; 5 to 12 years	None	Reduced Cooper test	None	None	Tennis ball throw; medicine ball (1kg) throw	None	None	Jumping a distance of 7m on two feet and on one foot	Standing broad jump	20m dash	10x5m shuttle run; Climbing up wall bars	None
IPFT (Bahrain); (31)	School and health; 9 to 19 years	Height; weight; %BF (skinfolds)	1-mile run/walk test	Handgrip	None	Back throw	None	None	None	None	None	4x10m shuttle run	None
INDARES (Czech Republic); (32)	School and health; 7 to 18 years	Height; weight; %BF (skinfolds); BIA	PACER; 1500m run / walk	None	Push-ups	Cricket ball throw	None	Modified curl-ups	Chair squats	None	60m dash	4x10m shuttle run	V sit and reach; shoulder stretch
NAPFA (Singapore); (33)	School and military; 12 to 24 years	None	1.5-mile run / walk	None	Pull-ups; flexed-arm hang (30 seconds)	None	None	Sit-ups with twist (1 minute)	None	Standing broad jump	None	4x10m shuttle run	Sit and reach
NFTP-PRC (China); (34)	School and health; 9 to 19 years	None	Shuttle run (50x8m); 4-, 3- and 2-minutes shuttle run (25m); Quarter-, half- and 1-mile run / walk; 1-minute jump rope	None	Bent arm hang; pull-ups; modified pull-ups; parallel-bars dips	None	None	Sit-ups	None	Standing broad jump	50m dash; 100m dash	4x10m shuttle run	None
NYPFP (USA); (35)	School, health, and military; 5 to 17 years	None	1-mile run / walk	None	Push-ups; modified push-ups; bent arm hang; pull-ups; modified pull-ups; parallel-bars dips	None	None	Sit-ups	None	Standing broad jump	None	None	None
NZFT (New Zealand); (36)	School and health; 6 to 12 years	Height; weight; %BF (skinfolds)	Cooper test (9 minutes)	None	None	Medicine ball throw; shot put (1 to 5kg); sand ball throw	None	Curl-ups	None	Standing broad jump	None	None	Sit and reach
PCPF (USA); (37)	School and health; 6 to 17 years	Height; weight; BMI; %BF (skinfolds)	PACER (20m and 15m), TAMT (aerobic behavior, level 1); 1-mile run/walk	None	Push-ups 90°; bent arm hang; pull-ups	None	None	Curl-ups; trunk lift	None	None	None	None	Sit and reach; shoulder stretch
PFAAT (Japan); (38)	School, sports, and health; 6 to 17 years	Height; weight; BMI	PACER	Handgrip	Pull-ups	Softball / handball throw	Back strength test	None	None	Vertical jump; standing broad jump	50m dash	Side-to-side steps	Sit and reach; stand and reach
PROESP (Brazil); (39)	School, sports, and health; 6 to 17 years	Height; weight; BMI; WC; height to waist ratio; wingspan	6-minutes run / walk	None	None	Medicine ball (2kg) throw	None	Sit-ups (1 minute)	None	Standing broad jump	20m dash	Square test (4x4m)	Sit and reach
Ready for Labor and Defense - GTO (Russia); (40)	School and military; 10 to 60 years	None	Running test (1 or 2 km); cycling (5km); cross-country running (0.5 to 1km)	None	Push-ups; pull-ups; rope climbing with legs	Tennis ball throw	None	None	None	Vertical jump; standing broad jump	30m, 50m, 60m, 80m or 100m dash	None	None
SLOfit (Slovenia); (41)	School and health; 6 to 19 years	Height; weight; %BF (skinfolds).	Half-mile run	None	Bent arm hang	None	None	Sit-ups (1 minute)	None	Standing broad jump	60m dash; 20-seconds plate tapping	Polygon backward	Stand and reach
UNIFITTEST (Czech Republic); (42)	School, sports, and health; 6 to 60 years	None	12-min run/walk	None	None	Medicine ball throw	None	Sit-ups (1 minute)	None	Standing broad jump	None	4 × 10m shuttle run	None
YMCA-YFT (USA); (43)	School and health; 6 to 17 years	Height; weight; %BF (skinfolds)	1-mile run / walk	None	Modified pull-ups	None	None	Curls-ups	None	None	None	None	None

*AAHPER, American Association for Health, Physical Education, and Recreation; AAUTB, Amateur Athletic Union Test Battery; ALPHA, Assessing Levels of Physical Activity and Fitness; ASSO-FTB, Adolescents and Surveillance System for the Obesity prevention – Fitness Test Battery; ACHPER, Australian Council for Health, Education and Recreation; CAHPER-FPT, Canadian Association for Health, Physical Education and Recreation-Fitness Performance Test II; CPAFLA, Canadian Physical Activity, Fitness and Lifestyle Approach; IPFT, International Physical Fitness Test; INDARES, International Database for Research and Educational Support; NAPFA, Singapore National Physical Fitness Award/Assessment; NFTP-PRC, National Fitness Test Program in the Popular Republic China; NYPFP, National Youth Physical Program; NZFT, New Zealand Fitness Test; PCPF, President's Challenge: Physical Fitness; PFAAT, Physical Fitness and Athletic Ability Test; PROESP, Projeto Esporte Brasil; USA, United States of America; YMCA-YFT, Young Men's Christian Association Youth Fitness Test*.

***Note:** Middle-body power and lower body strength are not shown in the table because no measurements / tests on these physical fitness components were presented in the analyzed physical fitness batteries*.

Most PF batteries (21 of 25) are exclusively for children and adolescents, while four of them are also extended to young adults (33) and adults (26, 40, 42). Also, even though all PF batteries can be performed in the school setting with the purpose of monitoring health-related indicators, some of them can be used in other settings such as sports and the army to assess physical performance. Two examples are the National Youth Physical Program from the United States Marines Youth Foundation (NYPFP) and the Ready for Labour and Defense (GTO) from Russia that is usually used to monitor PF for military purposes.

Regarding the PF components assessed in the batteries, only the CRF and the upper body strength, endurance and power were contemplated in all PF batteries. Middle-body and lower body strength, endurance and power were presented in most of the PF batteries, 21 of 25 and 20 of 25, respectively. Other components as agility (17 of 25) and body composition (16 of 25) were also contemplated in most PF batteries, although to a lesser extent. Flexibility (14 of 25) and speed (13 of 25) were the PF components less represented in the batteries, notwithstanding they were present in at least 50% of the identified PF batteries.

Among 25 identified PF batteries, a total of 87 PF tests, assessing the different PF components, were encountered. The PF component with the widest variety of different tests, that is, with 23, was CRF. It was followed by upper body strength, endurance and power with 21, speed with 10, middle-body strength and endurance with nine, body composition with eight, agility with seven, lower body endurance and power with five and flexibility with four different tests.

## Discussion

This systematic review provides a summary of existent PF batteries from around the world containing field-based health-related tests that can be performed by children and adolescents and used to monitor health status. A total of 25 different PF batteries from European, American, Asian, and Oceanian countries were identified. This knowledge can be useful for selecting standardized and validated PF tests and batteries, adjusted for the school setting and considering different PF components, and simultaneously, allows direct comparison between peers of the same age from different geographic locations.

Among children and adolescents, PF is associated with numerous health indicators, thus assessing PF has been suggested to be a reliable tool to monitor health in youth (1). Furthermore, PF batteries are considered a valid, simple, precise, and low-cost health monitoring tool (44). Given that in several countries, such as Australia, Bahrain, Brazil, Canada, Czech Republic, China, France, Italy, Japan, Norway, Portugal, New Zealand, Russia, Singapore, Slovenia, Spain, and the USA, the military, sport, health, and education sectors have been implementing and using PF batteries. Findings from this review corroborate the popularity of PF assessments, once 25 PF batteries from four different continents were identified.

Being a multi-component construct, examining PF as a whole, using only one or two tests is a misconception, as different associations between PF components and health indicators are observed (1, 45). Because of that, the existence of detailed PF batteries is of importance. Such batteries allow taking into account a cluster of PF tests that are validated for each PF component, and that together it is possible to monitor complementing indicators of health and vulnerability. In this review, body composition, CRF, and muscular fitness (MF) were identified as the components of PF most frequently assessed in PF batteries.

Assessing body composition is usually the result of different anthropometric measures and their relation, such as height, weight, or waist circumference, as well as methodologies to analyse the % of body fat, muscle mass, and hydration (44). The measures of body composition, used in PF batteries, identified in this review were BMI, waist circumference, % of body fat (skinfolds), height to waist ratio, waist to hip ratio, wingspan, and bioelectrical impedance analysis. Requiring only height and weight, the BMI is a non-invasive, inexpensive, practical, and a largely applicable anthropometric indicator of obesity (48, 49). On the other hand, BMI does not differentiate fat mass from lean mass and is thus an insufficient indicator of body fat or abdominal adiposity (50). In this line, to avoid misclassifications international experts have been suggesting waist circumference, which is a better indicator of central adiposity, as an alternative to BMI (50, 51). More precise measures of body composition, namely the % of body fat were also present in some batteries, assessed by skinfolds or bioelectrical impedance analysis. Skinfolds allow calculating the % of fat mass and fat-free mass, through specific equations and are a low-cost methodology but specific and intensive training is required to minimize potential measurement error (52). Bioelectrical impedance analysis is more precise and allows to examine the % of fat mass, muscle mass or hydration status, however, it requires specific equipment, individual calibration and is more difficult to operationalize (53).

The CRF is the most studied component of PF among children and adolescents (54), and not surprisingly was assessed in each of the PF batteries identified in this systematic review. Higher levels of CRF are associated with a lower risk of several health outcomes, namely obesity, cardiovascular diseases, and mental health (1). The importance of assessing CRF was also reflected in a large number of tests observed, and among these tests, the PACER and the 1-mile run/walk seemed to be present in the most PF batteries. Both, the PACER and 1-mile run /walk are widely validated and reliable for assessing the CRF in young populations (15, 55). From these test results, maximum aerobic capacity can be estimated. From all equations to estimate maximum aerobic capacity through these field-based PF tests, the equations proposed by Cureton et al. (56) for the 1-mile run/walk test and Barnet et al. (57) for the PACER had the strongest evidence of validity with Léger equation (56–59). However, recently some issues have been raised regarding the estimation of maximum aerobic capacity considering that a multitude of factors (e.g., sex, adiposity) have an influence, emphasizing that estimations should be carefully interpreted to avoid misconceptions (60–62). Also, using test results in terms of the number of laps, stages, or time may provide a clearer picture of the individual's CRF.

Muscular fitness, another important PF component, was also assessed in each of the PF batteries identified. However, different components of MF (i.e., upper body, middle-body and lower body strength, endurance and power, agility, speed, and flexibility) were assessed across the batteries. Similar to CRF, MF is also associated with several health outcomes in youth (45, 46). A total of 56 different tests to assess the several components of MF were identified. For the upper body, the most common tests were the handgrip, push-ups or bent arm hang test, which assessed endurance and power. Regarding the lower body, the standing broad jump and the vertical jump, both assessing power, were the most usual tests. Lastly, for the middle-body, curl-ups and sit-ups were the most common tests, assessing endurance. Most of these tests require minimum equipment and are easily applied within a school or class setting. Agility, speed, and flexibility were present in fewer PF batteries than the other components of muscular fitness. This may be because there is more evidence observing the associations of lower, upper, and middle body strength with health indicators (47).

A total of 25 PF batteries were identified in this systematic review and across them 87 different PF tests for body composition, CRF, and MF. A previous systematic review focused on PF tests indicated that the PACER (or 20-meter shuttle run), the handgrip strength and standing broad jump tests, the 4 × 10m shuttle run test, weight, BMI, skinfolds, circumferences, and % body fat estimated from skinfold thickness were the most reliable field-based PF tests for children and adolescents (63). In this review, the aforementioned tests are among the most used in the identified PF batteries, which also corroborates previous research on this topic (17). Notwithstanding, when selecting a measurement/ protocol test of body composition, CRF or MF to perform factors such as staff training, equipment cost and time should be considered, as they heavily influence data collection, validity, and feasibility. Also, to avoid data contamination and misinterpretations, all protocols should be clear and performed by trained personnel, such as physical education teachers and other specialists (44). Despite being beyond the scope of this paper, it is important to acknowledge that physical education, sport, and health professionals should have a pedagogical approach in the application of PF batteries. This means that the application of the PF batteries must be aligned with the promotion of meaningful, relevant, and positive experiences for children and adolescents (64).

This systematic review is not without some limitations. Firstly, the large number of articles and protocols for the same PF test may have resulted in an overlap of tests. Secondly, the terms selected to identify investigations and other documents describing the PF batteries, although highly thorough nevertheless may have excluded documents not matching the inclusion criteria. Also, the search was conducted in only five databases. Lastly, because of the different study designs and the integration of gray literature (not following a scientific structure, such as protocols) the risk of bias and study quality assessment was unfeasible. Yet, most importantly, the major strength of this review is the ample number of articles reviewed and time interval search, which resulted in the identification of a rich set of PF batteries from around the globe.

## Conclusion

The advances in the PF field-based assessment on school settings and health in youth resulted in the amplification of the number of existing batteries. On the one hand, diversity allows choosing the battery that most fits the specific purpose and setting of the assessment. On the other hand, it somehow complicates the comparability of data from different contexts, countries, or regions. Therefore, considering the connection between PF and health and the opportunity that the school setting provides to assess fitness in children and adolescents, we highlight the need for standardization and a consensus of PF assessments in this specific setting. In the European Union, a unique and actualized European PF battery would allow comparisons between European children and adolescents from different countries, to contribute to adequate and specific education and health public policies in the future.

## Data Availability Statement

The original contributions presented in the study are included in the article/supplementary material, further inquiries can be directed to the corresponding author/s.

## Author Contributions

AM and MP: conception and design and drafting the manuscript. DH-N, MP, JM, and FG: data acquisition. AM, SP, and BM: data analysis and interpretation. YD, AS, JM, DH-N, and AI: critical revision for intellectual content. DH-N and FG: administrative, technical or material support. All authors read and approved the final manuscript.

## Conflict of Interest

The authors declare that the research was conducted in the absence of any commercial or financial relationships that could be construed as a potential conflict of interest.
